# Religious Beliefs, Possession States, and Spirits: Three Case Studies from Sri Lanka

**DOI:** 10.1155/2012/232740

**Published:** 2012-08-30

**Authors:** Raveen Hanwella, Varuni de Silva, Alam Yoosuf, Sanjeewani Karunaratne, Pushpa de Silva

**Affiliations:** ^1^Department of Psychological Medicine, Faculty of Medicine, 08 Colombo, Sri Lanka; ^2^Finley Hospital, Finley, NSW 2713, Australia; ^3^University Psychiatry Unit (Ward 59), National Hospital of Sri Lanka, 08 Colombo, Sri Lanka

## Abstract

We describe three patients from different religious backgrounds in Sri Lanka whose possession states were strongly influenced by their religious beliefs. Patient A was a Buddhist who claimed to have special powers given by a local deity named Paththini. Patient B was a Catholic who experienced spirits around her whom she believed were sent by Satan. Patient C was a Muslim and believed she was possessed by spirits. The religious beliefs also influenced the help-seeking behaviour and the rituals or treatments to which they responded.

## 1. Introduction

Trance states are a detachment from ones' surroundings. Possession states are a replacement of the customary sense of personal identity by a new identity, attributed to the influence of a spirit, power, deity, or other person. They are associated with stereotyped “involuntary” movements or amnesia and classified as dissociative phenomena [1].

Possession states have been described in almost all societies of the world. In a global survey of 488 societies, 437 (90 percent) had one or more institutionalized, culturally patterned form of altered states of consciousness. In 252 societies, such experiences were attributed to possession [2]. In most cultures possessing agents were thought to be spirits of deceased individuals, deities, animals, or devils. However the identity of these appears to depend on the culture [3].

Sri Lanka comprises several religious groups. The majority are Buddhists (76.7%) followed by Muslims (8.4%), Hindus (7.7%), and Christians (7%) [4].

We describe 3 patients whose psychopathology was influenced by their religious and cultural beliefs. Two patients were diagnosed with trance and possession states and one with an acute stress reaction.

## 2. Case Presentation

Patient A was a 34 year old fortune teller a mother of 2 children and a Buddhist by religion resident in a rural area. Around the age of 18 years she claimed she could communicate with a local deity named “Paththini”. She then began fortune telling and healing people with the power given to her by the Goddess Paththini. This was a source of income for her. This behaviour was not considered problematic or abnormal by her, the family or the community.

Three months prior to presentation to psychiatric services she began recalling her past births during trance states. She conversed with Goddess Paththini during the trance states and recalled details after coming out of the trance. The trance states were induced by religious rituals. She described her own previous lives and those of her family members and attributed various current behaviour patterns to consequences of acts done during past lives. She identified an acquaintance as her lover in a previous birth and even introduced him to her husband. She frequently conversed with him over the telephone which resulted in family conflicts. This led to the family seeking help from a psychiatrist.

Patient B was a 21-year-old single unemployed female of Muslim religious background from an urban area. Ten months before contact with psychiatric services, she started developing possession states. At the beginning these occurred about once a day but increased in frequency to 4-5 times a day during the last month. The patient and the family believe that she is possessed by demons. While possessed her eyes opened wide, she stretched out her hands, and screamed loudly in anger. These states lasted for about 10 minutes. She was following an accountancy course and the family identified stressors associated with her education as the reason for the possession state. Epilepsy was considered in the differential diagnosis of this patient because of the episodic nature of the illness.

Patient C was a 66-year-old housewife, a mother of 4 children and a Catholic by religion resident in a semiurban area. Four months previously she has attended the funeral of a known person who had died in a road traffic accident. Following this she began seeing images of the funeral and of the dead person coming out of the coffin towards her. She was extremely distressed by it. She was generally fearful of attending funerals and avoided them whenever possible. She began seeing frightening images of figures with wide eyes. She believed these were demons or evil spirits sent by Satan to check on her. She did not go into a trance state and/or believe she was possessed by the devil but believed that Satan and evil spirits were around her.

## 3. Discussion

All 3 patients' symptoms could be explained psychodynamically and the psychopathology was strongly influenced by their religious beliefs. The patients were all Sri Lankans, but their different religious beliefs influenced their psychopathology and the understanding of their illness by the families. All 3 patients engaged in religious rituals and exorcism ceremonies to get rid of the possession states or disturbing experiences but when there was no improvement sought help from a psychiatrist. 

Buddhism does not believe in a God but many Buddhists in Sri Lanka worship Hindu Gods and other Gods indigenous to Sri Lankan culture. God “Paththini” is one such. Buddhists believe in rebirth and sometimes children claim to recall their past births. For patient A, her family and the villagers strongly believed that people with special powers could predict the future. Her possession states were for many years adaptive as she earned a living through fortune telling as a result of special powers acquired through possession states. Later on she began recalling her past births. This too is in keeping with her religious beliefs. Her family and the community believed that the descriptions of her past lives and those of others to be true. Therefore we see that the content of psychopathology is strongly influenced by the patients' cultural and religious beliefs and these beliefs are also shared by others in the community. Recent trance states and recall of previous births had an underlying motive of justifying her relationship with a male. Because of the religious and cultural sanction, she was able to continue this extramarital relationship with knowledge of her husband and the community. Whether this behaviour was fully conscious or not is open to debate.

Patient B was of Islamic faith and people of Islamic faith believe in one God called “Allah.” God alsocreated a system to take his orders and messages to humans, especially to his nominated prophets. God created 124 000 prophets beginning from Adam and the last prophet is the superior of all, Muhammad. Allah created his messengers from “light” known as Angel's alias “Malaks.” Allah also created other creatures known as “Jinn.” They live in a parallel world to humans. They are made of energy such as “light/fire.” There are numerous references to Jinn in the Qur'an and Hadith. According to Islam, Jinn live alongside other creatures but form a world other than that of mankind. According to Islamic writings true Jinn possession can cause a person to have fitsand to speak in an incomprehensible language, and so forth. The possessed is unable to think or speak from his will.In case of real possession some faith-healers have the power to expel Jinn from the possessed by reciting verses from the Quran. In Sri Lanka, the culture of Muslims may also be influenced by the Hindu and Buddhism beliefs.

Patient C was a Catholic and her experiences was of Satan again influenced by her religious beliefs and shared by others in the community. Her acute anxiety and distress manifested as spirits which was influenced by her belief that the dead can appear as spirits. The Christian New Testament includes exorcism among the miracles performed by Jesus. Christians believe in Demonic possession and Satan the devil. Exorcism is still a recognized practice of Catholicism.

Patient A was symptom free after a period of stay in an inpatient unit. During this period she was given brief insight-oriented psychotherapy and couple therapy was started with a view to improving her relationship with her husband. However there were major incompatibilities between them which could not be resolved. It is doubtful that she would be willing to give up the cultural sanctions which would enable her to continue her extramarital relationship without censure. Patient B dropped out from psychiatric services after the initial contact and sought the help of religious rituals. She subsequently presented for a follow-up visit and had not experienced possession states for 3 months. The patient and family were of the opinion that her “cure” was due to these religious rituals. Patient C recovered fully after a short period of stay in an inpatient unit where she was treated with anxiolytics and supportive psychotherapy.

This paper highlights that possession agents described as spirits, powers, or deities have detailed identities among communities often influenced by religious beliefs. These beliefs also influence the help-seeking behaviour and the rituals or treatments to which they respond. It also highlights that common cultural beliefs shared by Sri Lankans are incorporated into the religious beliefs and practises by the different religious groups.
